# Anti-Tumor Effect in Human Lung Cancer by a Combination Treatment of Novel Histone Deacetylase Inhibitors: SL142 or SL325 and Retinoic Acids

**DOI:** 10.1371/journal.pone.0013834

**Published:** 2010-11-04

**Authors:** Shaoteng Han, Takuya Fukazawa, Tomoki Yamatsuji, Junji Matsuoka, Hiroyuki Miyachi, Yutaka Maeda, Mary Durbin, Yoshio Naomoto

**Affiliations:** 1 Department of Gastroenterological Surgery, Graduate School of Medicine, Dentistry and Pharmaceutical Sciences, Okayama University, Okayama, Japan; 2 Division of Pharmaceutical Sciences, Graduate School of Medicine, Dentistry and Pharmaceutical Sciences, Okayama University, Okayama, Japan; 3 Department of General Surgery, Kawasaki Medical School, Okayama, Japan; 4 Department of Hepatobiliary Surgery, Shengjing Hospital, China Medical University, Shenyang, China; 5 Division of Pulmonary Biology, Cincinnati Children's Hospital Medical Center, Cincinnati, Ohio, United States of America; 6 Department of Ecology and Evolutionary Biology, University of California Irvine, Irvine, California, United States of America; University of Giessen Lung Center, Germany

## Abstract

Histone deacetylase (HDAC) inhibitors arrest cancer cell growth and cause apoptosis with low toxicity thereby constituting a promising treatment for cancer. In this study, we investigated the anti-tumor activity in lung cancer cells of the novel cyclic amide-bearing hydroxamic acid based HDAC inhibitors SL142 and SL325. In A549 and H441 lung cancer cells both SL142 and SL325 induced more cell growth inhibition and cell death than the hydroxamic acid-based HDAC inhibitor suberoylanilide hydroxamic acid (SAHA). Moreover, the combination treatment using retinoid drugs ATRA or *9-cis* RA along with SL142 or SL325 significantly induced more apoptosis and suppressed colony formation than the single use of either. The expression of the retinoic acid receptors RARα, RARβ, RXRα and RXRβ were unchanged with the treatment. However a luciferase reporter construct (pGL4. RARE 7x) containing seven tandem repeats of the retinoic acid responsible element (RARE) generated significant transcriptional activity after the combination treatment of retinoic acids and SL142 or SL325 in H441 lung cancer cells. Moreover, apoptosis-promoting Bax expression and caspase-3 activity was increased after the combination treatment. These results suggest that the combination treatment of SL142 or SL325 with retinoic acids exerts significant anti-tumor activity and is a promising therapeutic candidate to treat human lung cancer.

## Introduction

Histone deacetylase (HDAC) and histone acetyltransferase (HAT) are involved in the co-regulation of chromatin remodeling and the functional regulation of gene transcription [1]. HDACs regulate various kinds of biological processes, including proliferation, differentiation, and apoptosis [2]. There are several reports that altered HAT or HDAC activity is associated with various cancers [3], [4], [5], [6]. A number of small-molecule HDAC inhibitors have been developed as anti-cancer agents. In fact, HDAC inhibitors were shown to induce cell cycle arrest, differentiation and apoptosis in a variety of malignant cells. HDAC inhibitors increase acetylation of histones and transcription factors, which can reverse gene silencing thus facilitating gene expression [7]. These effects are mediated in part by selective alteration in gene expression, such as the induction of p21waf expression [8]. However, not all genes are up-regulated by treatment with HDAC inhibitors, and the ratio of up-regulated to down-regulated genes has been found to be close to 1∶1 [9].

Lung cancer is the leading cause of death worldwide [7]. The two main forms of lung cancer are non–small cell lung cancer (NSCLC) and small cell lung cancer (SCLC). Treatment outcomes for advanced NSCLC using chemotherapeutic agents have been disappointing. Clearly, further investigation is urgently needed for the treatment of advanced NSCLC. New treatments with novel mechanisms of action, including agents that target angiogenesis and the regulation of gene expression by retinoic acids have been explored [10], [11], [12], [13]. Without ligand, retinoic acids receptors act as transcriptional repressors due to the binding of corepressor complexes that contain histone deacetylases (HDAC). Ligand binding releases these co-repressors and recruits co-activator complexes, which could generate histone acetylase activity [13], [14]. It has been reported that the combinations of all-trans retinoic acid and HDAC inhibitors have an anti-tumor effect [15], [16]. The combination of all-*trans* retinoic acid (ATRA) and some HDAC inhibitors showed an anti-tumor effect in neuroblastoma [15], [16]. The combination therapy of retinoic acids with HDAC inhibitors may improve efficacy while reducing side effects The purpose of the present study is to develop a new strategy to treat lung cancer. We have therefore analyzed the effect of using the combination of novel, class selective cyclic amide-bearing hydroxamic acid based HDAC inhibitors SL142 or SL325 [17] combined with retinoic acids to test their efficacy for treating lung cancer.

## Materials and Methods

### Reagents

**Figure 1:**
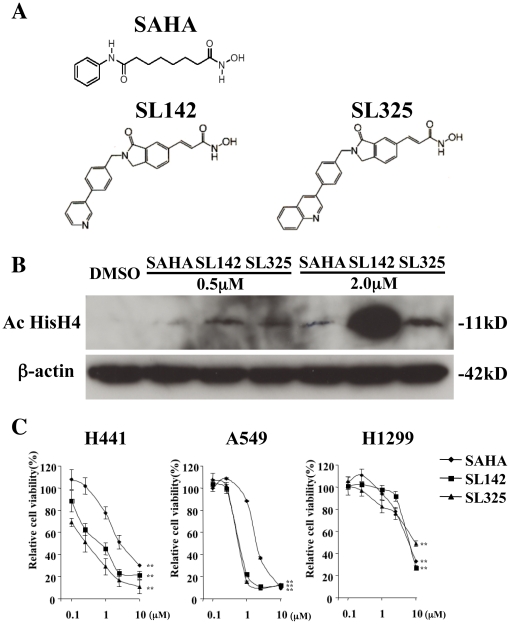
SL142 and SL325 significantly suppressed cell viability in H441 and A549 lung cancer cells. **A**. Chemical structure of SAHA, SL142 and SL325. **B**. Detection of H4 acetylation by immunoblot 24 hours after SAHA, SL142 or SL325 treatment (0.5 or 2.0 µM) in H441 lung cancer cells. β-actin is shown as control. **C**. Effect on cell viability induced by SAHA, SL142 or SL325. Cells were plated in 96-well plates at a density of 1×10^3^ cells/well 24 hours prior to treatment with SAHA, SL142 or SL325 (0.1 to 10 µM). Cell viability was evaluated at 96 hours following treatment by the WST1 assay (Roche, Basel, Switzerland) according to the manufacturer's protocol. **, significant difference from the cell viability treated with 0.1 µM of SAHA, SL142 or SL325 (p<0.01).

SL-142 ((E)-3-(2-(4-pyridin-4-yl)benzyl-1-oxoisoindolin-6-yl)-N-hydroxyacrylamide) and SL-325 ((E)-3-(2-(4-quinolin-3-yl)benzyl-1-oxoisoindolin-6-yl)-N-hydroxy-acrylamide) are novel isoindolinone-hydroxamic acid based histone deacetylase (HDAC) inhibitors derived from our structural development studies of the multi-drug template thalidomide for the creation of structurally novel drugs (Fig. 1A)[18], [19].

### Cell Lines and Culture Conditions

The human non-small cell lung cancer cells A549, H441 and H1299 were obtained from the American Type Culture Collection (Manassas, VA) and grown in Ham's F12 (A549 cells), RPMI 1640 (H1299 cells) with high glucose Dulbecco's modified Eagle medium supplemented with 10% heat-inactivated fetal bovine serum. All cell lines were cultured in 10% CO_2_ at 37°C. The lysates of human adult lung tissue were obtained from Novas Biologicals (Littleton, CO).

### Immunoblot analysis

Cells were lysed in ice cold lysis buffer [1% Triton X-100, 20 mM Tris-HCL (pH 8.0), 137 mM NaCl, 10% glycerol (v/v), 2 mM EDTA, 1 mM sodium orthovanadate (v/v) 1 mM phenylmethylsulfonyl fluoride]. Cell lysates were clarified by centrifugation (10 min at 15,000× g at 4°C) and protein concentration was determined using the DC protein assay (BioRad, Hercules, CA). Equal amounts of protein were separated on an SDS-PAGE gel. The gel was electrophoretically transferred to a Hybond PVDF transfer membrane (Amersham, Arlington Heights, IL). The membrane was incubated with primary and secondary antibodies according to the Supersignal^R^ West Pico chemiluminescence protocol (Pierce, Rockford, IL) to detect secondary antibody binding. Antibody specific for β-actin antibody was obtained from Sigma (St. Louis, MO) and antibody specific for human RARα and RXRα were obtained from Santa Cruz Biotechnology (Santa Cruz, CA). Anti- RARβ, RXRβ and Histone H4 acetyl antibody were obtained from Abcam (Cambridge, UK). Antibody specific for human Bax, p21 and p27 were obtained from Cell Signaling Technology (Danvers, MA). Secondary horseradish peroxidase-conjugated antibodies were obtained from Jackson Immunoresearch Laboratories (West Grove, PA).

### Inhibition and measurement of caspase activity

Caspase-3 activity was determined using ApoAlert caspase colorimetric assay kits obtained from Clontech (Mountain View, CA) according to the manufacturer protocol. The fold increase in the activity was determined by comparing the levels of caspase activity in treated cells with those in control cells (DMSO). For caspase inhibition, cells were incubated with 100 µM zVAD-fmk obtained from BioVision (Mountain View, CA).

### Flow cytometric analysis for apoptosis

Cells were plated in 24-well plates at a density of 0.5–1.0×10^5^ cells per well 1 day before the treatments. After 72 or 96 hours, cells were harvested and washed once with PBS. Cells were resuspended in PBS containing 0.2% Triton X-100 and 1mg/ml RNase for 5 min at room temperature and then stained with propidium iodide at 50 µg/ml to determine sub-G_0_/G_1_ DNA content using a FACScan. Doublets, cell debris, and fixation artifacts were gated out, and sub-G_0_/G_1_ DNA content was determined using Cell Quest Ver. 3.3 software.

### Clonogenic survival

H441 cells were first plated at a density of 2×10^5^ cells per well in six-well plates 24 hr before treatment. After treatment with HDAC inhibitors (0.5 µM) and/or retinoic acids (2.5 µM) for 36 hours, cells were released from the dish by incubation with trypsin/EDTA, counted, and plated in triplicate at a density of 2×10^3^ cells in 100-mm dishes for 16 days. The cells were then fixed with 100% methanol and allowed to air dry. The cells were stained with 0.1% crystal violet. Colonies (groups of aggregated cells numbering at least 50) were then counted. The mean number of the PBS treated cells was arbitrarily set to 100%, and all other numbers were normalized accordingly.

### Plasmids and Transient transfection reporter assays

Double stranded DNA containing seven repeats of the RARE sequence [20], [21] was synthesized and ligated into the NheI and HindIII sites of the pGL.4.24 luciferase reporter construct (pGL4. RARE 7x; Promega, Madison, WI). The luciferase reporter construct pGL4 Bax was generated by subcloning the promoter region of Bax −1570/+68 from genomic DNA using PCR primers: 5′-tttctcgag^XhoI^gaagtccaagagatcttcctgacaccctagtctga and 5′- tttaagctt^HindIII^ caccgccgctcccgccgccgcctctcgccgggtcc. The PCR-generated fragment was digested with XhoI and HindIII and subcloned directly into the pGL.4.24 - luciferase reporter construct (Promega). All transfections were carried out in six-well plates. H441 lung cells were seeded 24 hours prior to transfection at a density of 0.3×10^6^/well. Transfections were carried out with Lipofectin (Invitrogen Life Technologies, Carlsbad, California, USA) in accordance with the manufacture's protocol. H441 lung cells were seeded 24 hours prior to transfection at a density of 0.3×106/well. Transfections were carried out with Lipofectin (Invitrogen Life Technologies, Carlsbad, California, USA) in accordance with the manufacture's protocol. 4 hours after transfection, cells were treated with HDAC inhibitors and/or retinoic acids. 24 hours after HDAC inhibitors and/or retinoic acids treatment, cells were harvested. The results of one representative experiment are presented as fold induction of relative light units normalized to β-galactosidase activity relative to that observed for the control vectors. Each experiment was repeated at least three times. Error bars indicate the standard deviation from the average of the triplicate samples in one experiment.

### Statistical analysis

Statistically significant differences between means and medians of studied groups were evaluated using Student's t-test or the non-parametric Mann-Whitney U-test. An analysis of variance (ANOVA) test, where appropriate, was used to identify statistical significance for multiple comparisons. Statistical significance was defined as p<0.05 (*, #), p<0.01 (**).

## Results

### Effects of SL142 and SL325 on H4 acetylation

To analyze the histone deacetylase activity of SL142 and SL325, immunoblot analysis was performed. Incubation of H441 cells for 24 hours with SAHA, SL142 and SL325 caused a dose-dependent increase in histone H4 acetylation. Both SL142 and SL325 demonstrated stronger H4 acetylation than SAHA at concentrations of 0.5 µM and 2.0 µM. SL142 caused a much greater increase in histone H4 acetylation than SL325 at a concentration of 2.0 µM (Fig. 1B). These results demonstrate that both SL142 and SL325 increase histone H4 acetylation more than SAHA [22] in H441 lung cancer cells.

### SL142 and SL325 significantly suppressed cell viability in H441 and A549 lung cancer cells

In order to analyze the anti-tumor effect of HDAC inhibitors, we measured cell viability by the MTS assay after treatment with SAHA, SL142 and SL325 in H441, A549 and H1299 lung cancer cells. The cell viability assay revealed that all the HDAC inhibitors (SAHA, SL142 and SL325) strongly suppressed cell viability of H441, A549 and H1299 lung cancer cells at a concentration of 10 µM. In lower concentrations (0.5–5.0 µM), both SL142 and SL325 significantly suppressed the cell viability of H441 and A549 lung cancer cells (Fig. 1C). These results suggest that SL142 and SL325 are more effective in suppressing the cell viability of H441 and A549 lung cancer cells than SAHA and at lower concentrations.

### SL142 and SL325 significantly increased caspase-3 activity induced apoptosis in H441 and A549 lung cancer cells

**Figure 2:**
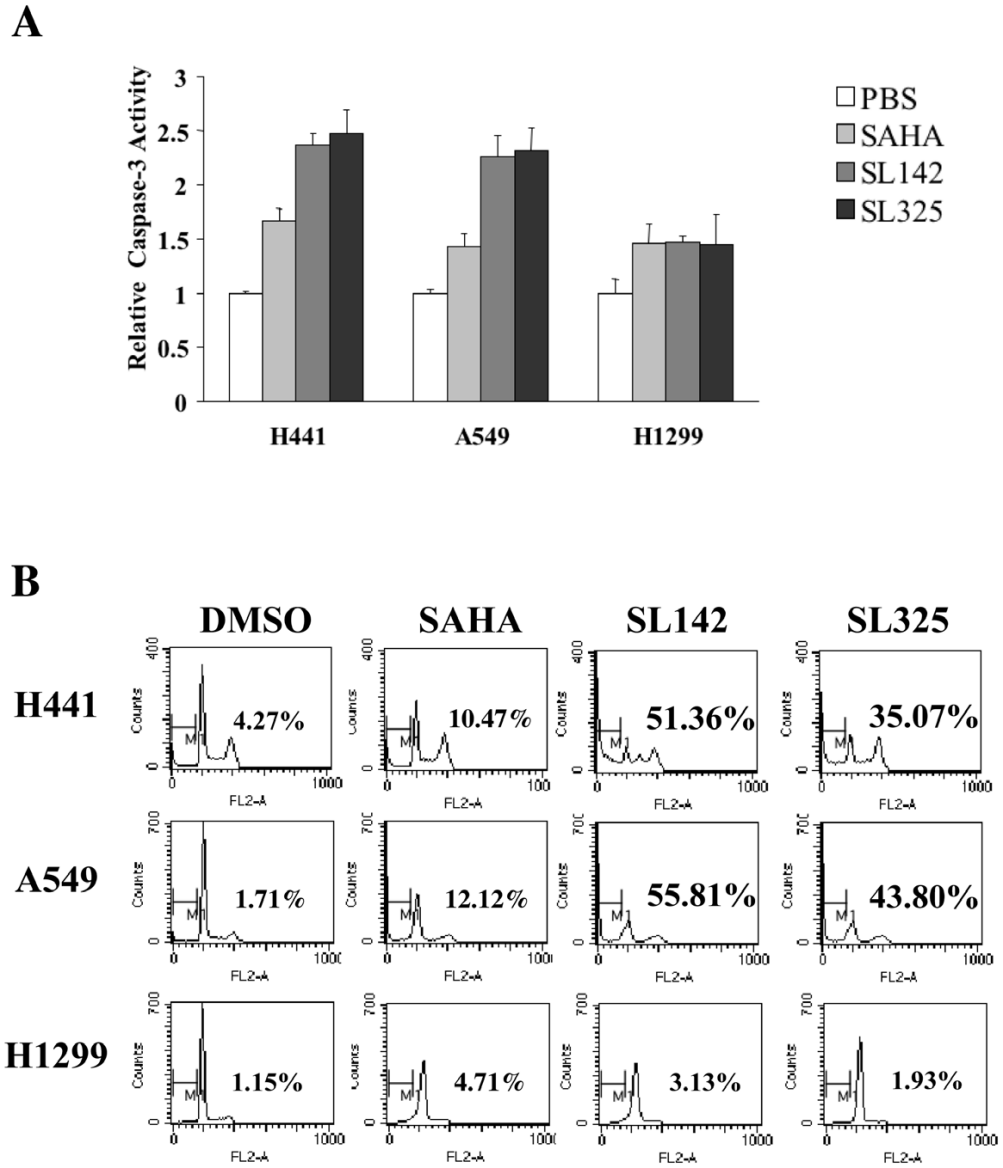
SL142 and SL325 suppressed cell growth and induced apoptosis in H441 and A549 lung cancer cells. **A**. Analysis of caspase-3 activity in A549, H441 and H1299 lung cancer cells after SAHA, SL142 and SL325 treatment. Cells were treated with SAHA, SL142 or SL325 at a concentration of 2.5 µM for 36 hours. Triplicate experiments were performed; data represent the mean-fold increase ± S.E. Significant difference from cells treated with PBS and cells treated with HDAC inhibitors indicated was shown (*p<0.05, **<0.01). **B**. Flow cytometric analysis of apoptosis induced by SAHA, SL142 or SL325. Cells were infected with SAHA, SL142 or SL325 at the concentration of 2.5 µM for 72 hours and sub-G_0_/G_1_ DNA content was measured by propidium iodide staining and flow cytometric analysis.

We analyzed caspase-3 activity and apoptosis in H441, A549 and H1299 lung cancer cells after treatment with HDAC inhibitors. SAHA, SL142 and SL325 increased caspase-3 activity in all cell types. Significantly, SL142 and SL325 induced higher caspase-3 activity (2.25– to 2.48 fold) than SAHA (1.42– to 1.66 fold) in H441 and A549 lung cancer cells (Fig. 2A). Next we investigated apoptosis induced by HDAC inhibitors in lung cancer cells. As shown in Fig. 2B, SL142 and SL325 induced more sub-G_0_/G_1_ DNA content (51.36–55.81% and 35.07–43.80% respectively) than SAHA (10.47–12.12%) in H441 and A549 lung cancer cells after a 72 hour treatment. However, these HDAC inhibitors induced less apoptosis in H1299 lung cancer cells. These results suggest that SL142 and SL325 more effectively increase caspase-3 activity and induce more apoptosis in H441 and A549 lung cancer cells than SAHA.

### ATRA or *9-cis* RA enhanced caspase-3 activity induced by SL142 or SL325 and increased apoptosis in H441 pulmonary adenocarcinoma cells

**Figure 3:**
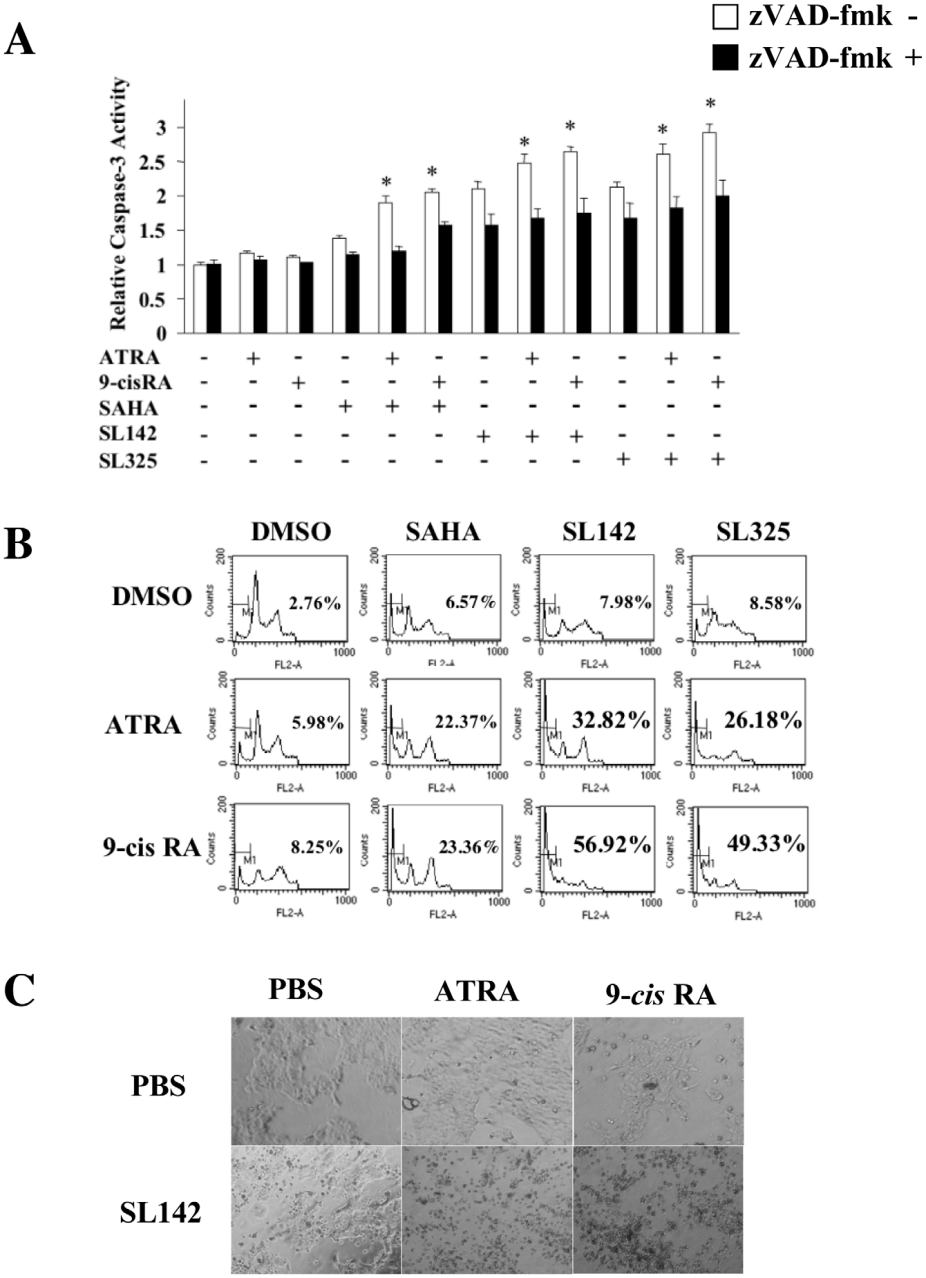
Combination treatment of retinoic acids and SL142 or SL325 more significantly induced cell death in H441 lung cancer cells than those of single use. **A**. Analysis of caspase-3 activity induced by ATRA or *9-cis* RA (2.5 µM) and/or SAHA, SL142 or SL325 (0.5 µM) in H441 lung cancer cells. Cells were treated with SAHA, SL142 or SL325 at the concentration of 2.5 µM for 36 hours. After 36 hours of treatment, sub-G_0_/G_1_ DNA content was measured by propidium iodide staining and flow cytometric analysis. Triplicate experiments were performed; data represent the mean-fold increase ± S.E. *, significant difference from control cells (cells without treatments) (p<0.05). **B**. Flow cytometric analysis of apoptosis induced by ATRA or *9-cis* RA (2.5 µM) and/or SAHA, SL142 or SL325 (0.5 µM) in H441 lung cancer cells. After 96 hours of treatment, sub-G_0_/G_1_ DNA content was measured by propidium iodide staining and flow cytometric analysis. **C**. Morphological analysis of H441 cells after ATRA or *9-cis* RA (2.5 µM) and/or SL142 (0.5 µM) treatment.

To analyze the combination effect of retinoic acids with SL142 or SL325, we examined the caspase-3 activity and sub-G_0_/G_1_ DNA content using propidium iodide staining with flow cytometry and caspase-3 activity after single or combination treatment. As shown in Fig. 3A, both ATRA and *9-cis* RA induced no caspase-3 activity while a single treatment of SAHA, SL142 or SL325 significantly increased caspase-3 activity (1.38– to 2.13-fold) in H441 pulmonary adenocarcinoma cells. Importantly, retinoic acid enhanced caspase-3 activity induced by SAHA, SL142 or SL325 and the combination of ATRA or *9-cis* RA, and SL142 or SL325 increased caspase-3 activity (2.47– to 2.91-fold) more than ATRA or *9-cis* RA and SAHA alone (1.90– to 2.04-fold). 100 µM of zVAD-fmk effectively inhibited the caspase-3 activity in all the experiments. Next, we investigated the amount of apoptosis induced by the HDAC inhibitors in lung cancer cells. Sub-G_0_/G_1_ DNA content was increased to 5.98% to 8.58% after a single treatment of retinoic acids or HDAC inhibitors. However combination treatment of SAHA and ATRA or *9-cis* RA increased sub-G_0_/G_1_ DNA content to 22.37% and 23.36% respectively. Moreover, SL142 and ATRA or *9-cis* RA increased sub-G_0_/G_1_ DNA content to 32.82% and 56.92% and SL325 and ATRA or *9-cis* RA increased the content to 26.18% and 49.33% respectively (Fig. 3B). The sub-G_0_/G_1_ DNA content induced by the single or combination treatment in normal human lung fibroblast (NHLF) was less than H441 cells (data not shown). Morphological analysis showed that more cell death was induced after the combination treatment of SL142 and ATRA or *9-cis* RA than those of single use (Fig. 3C). These results suggest that ATRA or *9-cis* RA increase caspase-3 activity and apoptosis induced by SAHA, SL142 or SL325.

### Effects of combination treatment of retinoic acids and SL142 or SL325 on suppression of colony formation of H441 lung cancer cells

**Figure 4:**
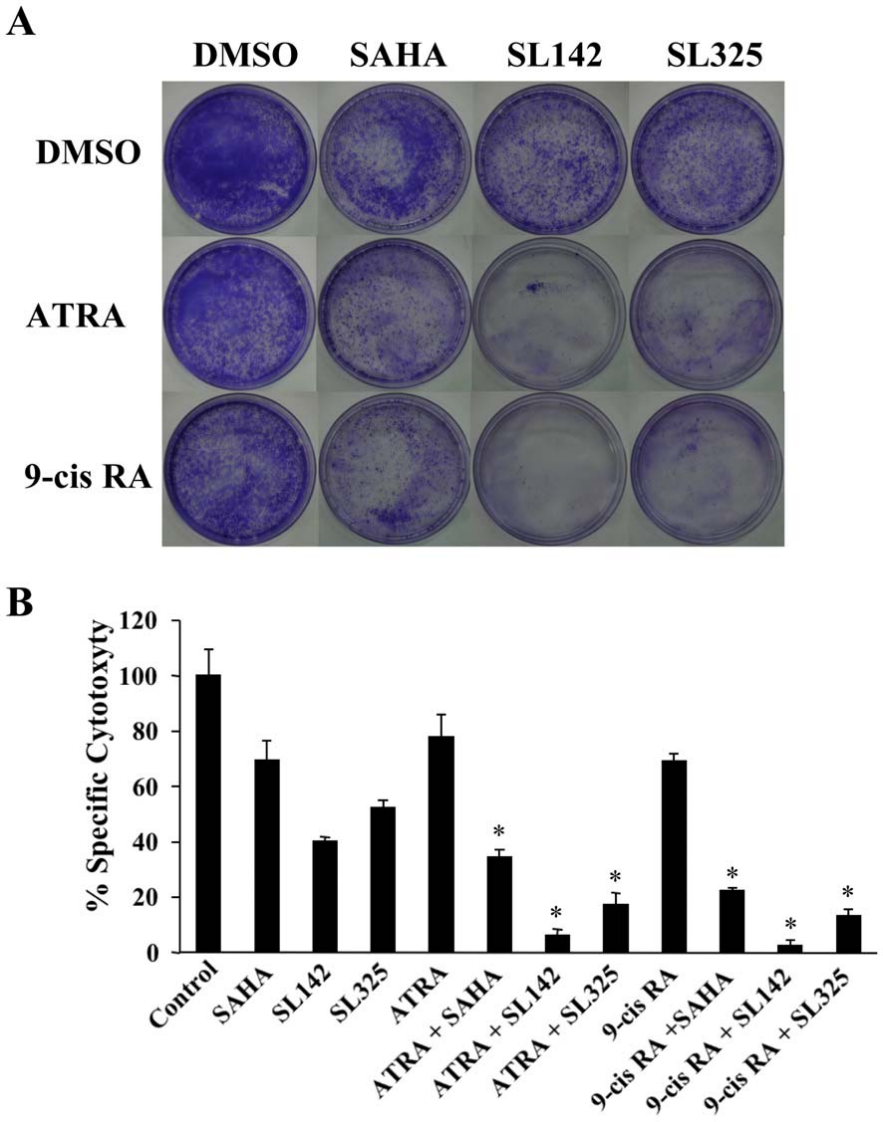
Combination effects of ATRA or *9-cis* RA and SAHA, SL142 or SL325 on suppression of colony formation in H441 lung cancer cells. **A**. Colony formation of H441 cells treated with ATRA or *9-cis* RA (2.5 µM) or/and SAHA, SL142 or SL325 (0.5 µM) for 36 hours. The assay was initiated by plating 2×10^3^ cells per 100-mm dishes. After 16 days, cells were fixed and stained with crystal violet. Representative pictures of experiments performed in triplicate are shown. **B**. Mean colony numbers were derived from quantitation of triplicate dishes for each treatment and percentage-specific cytotoxicity compared to colony formation in the DMSO group (control) was calculated. *, p<0.05 versus single agents.

To analyze the antitumor effect of the combination treatment of ATRA or *9-cis* RA and SL142 or SL325, a clonogenic assay was performed. As shown in Fig. 4A and 4B, ATRA or *9-cis* RA weakly suppress colony formation of H441 lung cancer cells (69.1–78.0%), while SAHA, SL142 or SL325 more strongly suppress colony formation (40.2–69.4%). Significantly, ATRA or *9-cis* RA enhanced the anti-tumor effect of SAHA, SL142 or SL325 especially when combined with SL142 or SL325 and ATRA or *9-cis* RA drastically suppressed colony formation (2.52–13.22%). These results suggest that the combination treatment of SL142 or SL325 and ATRA or *9-cis* RA synergically inhibits colony formation of H441 lung cancer cells.

### The combination treatment of SL142 or SL325 and ATRA or *9-cis* RA synergically increases the transcriptional activity of RARE but does not alter expression of RAR and RXR

**Figure 5:**
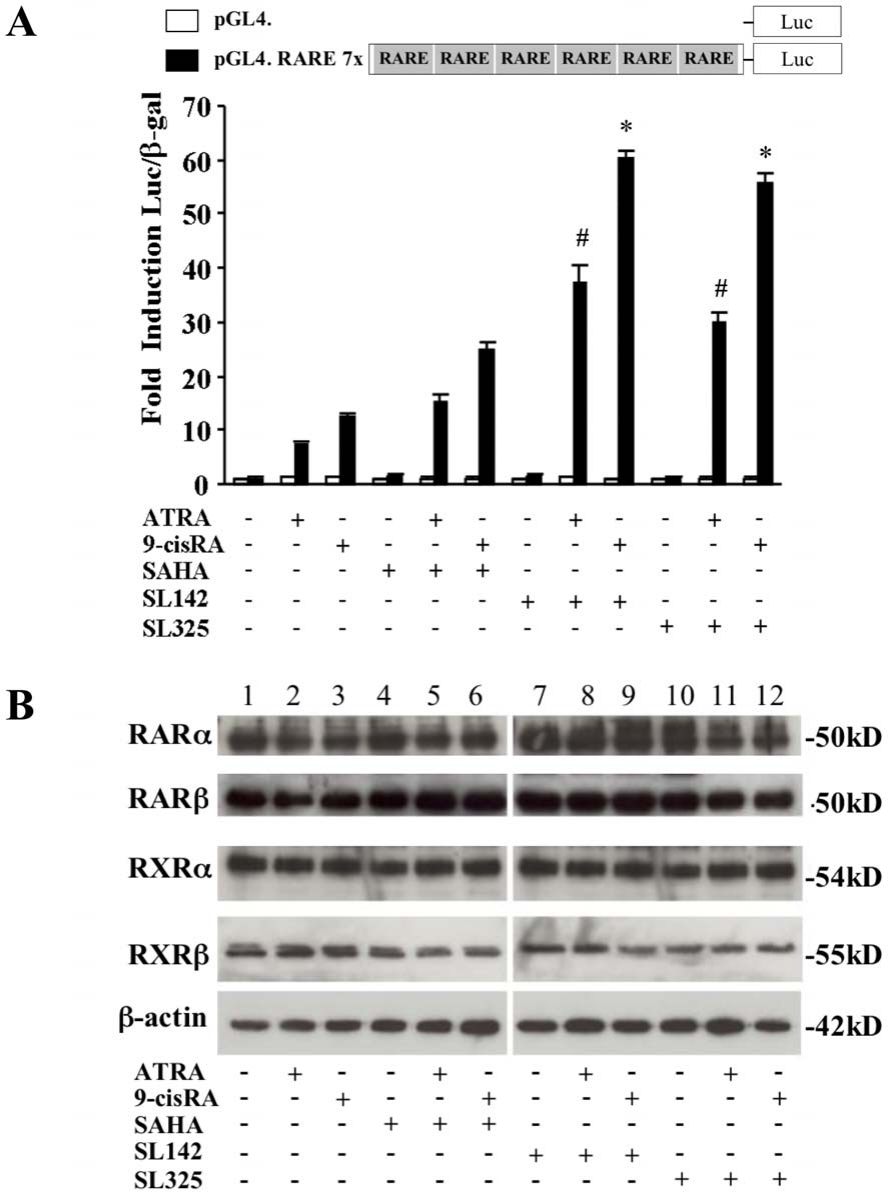
The combination treatment of retinoic acids and/or SL142 or SL325 synergically increased the transcriptional activity of RARE in H441 lung cancer cells. **A**. Transient transfection reporter assays in H441 cells with pGL4. or pGL4.RARE 7x (2 µg), plus pCMV. β-galactosidase (2 µg) after retinoic acids (2.5 µM) and/or SL142 or SL325 (0.5 µM) retinoic acids treatment. Results are presented as fold induction of relative light units normalized to β-galactosidase activity relative to that observed for control constructs. ^#^, significant difference from the promoter activity generated by pGL4.RARE 7x after ATRA and SAHA treatment (p<0.05). *, significant difference from the promoter activity generated by pGL4.RARE 7x after *9-cis* RA and SAHA treated cells (p<0.05). **B**. Immunoblot analysis of RARα, RARβ, RXRα and RXRβ expressions after retinoic acids and/or SL142 or SL325 treatment in H441 lung cancer cells. Human lung cancer cells were harvested 24 hours after treatment with ATRA or *9-cis* RA (2.5 µM) or/and SAHA, SL142 or SL325 (0.5 µM). β-actin is shown as control.

To analyze the transcriptional activity of the retinoic acid responsive element (RARE) after the treatment of SL142 or SL325 and ATRA or *9-cis* RA, we generated a luciferase construct driven by seven tandem repeats of the RARE element: pGL4. RARE 7x [20], [21]. pGL4. RARE 7x generated significant promoter activity (5.26– to 17.11-fold) after administration of 2.5 µM ATRA or *9-cis* RA treatments in H441 lung cancer cells. However, the construct showed little transcriptional activity after 0.5 µM of SAHA, SL142 or SL325 treatment in the cells (1.26– to 1.43- fold). Importantly, SL142 and SL325 more significantly enhanced the promoter activity of RARE after treatment of ATRA or *9-cis* RA. (29.94– to 37.42-fold, 55.58– to 60.14-fold respectively) compared to SAHA (14.99– to 24.65-fold) (Fig. 5A). However, as shown in Fig. 5B, immuno blot analysis demonstrated that little change was seen in the expression of RARα RARβ, RXRα and RXRβ expression after the single or combination treatment in H441 lung cancer cells.

### The combination treatment with SL142 or SL325 and ATRA or *9-cis* RA increases Bax expression in H441 lung cancer cells

**Figure 6:**
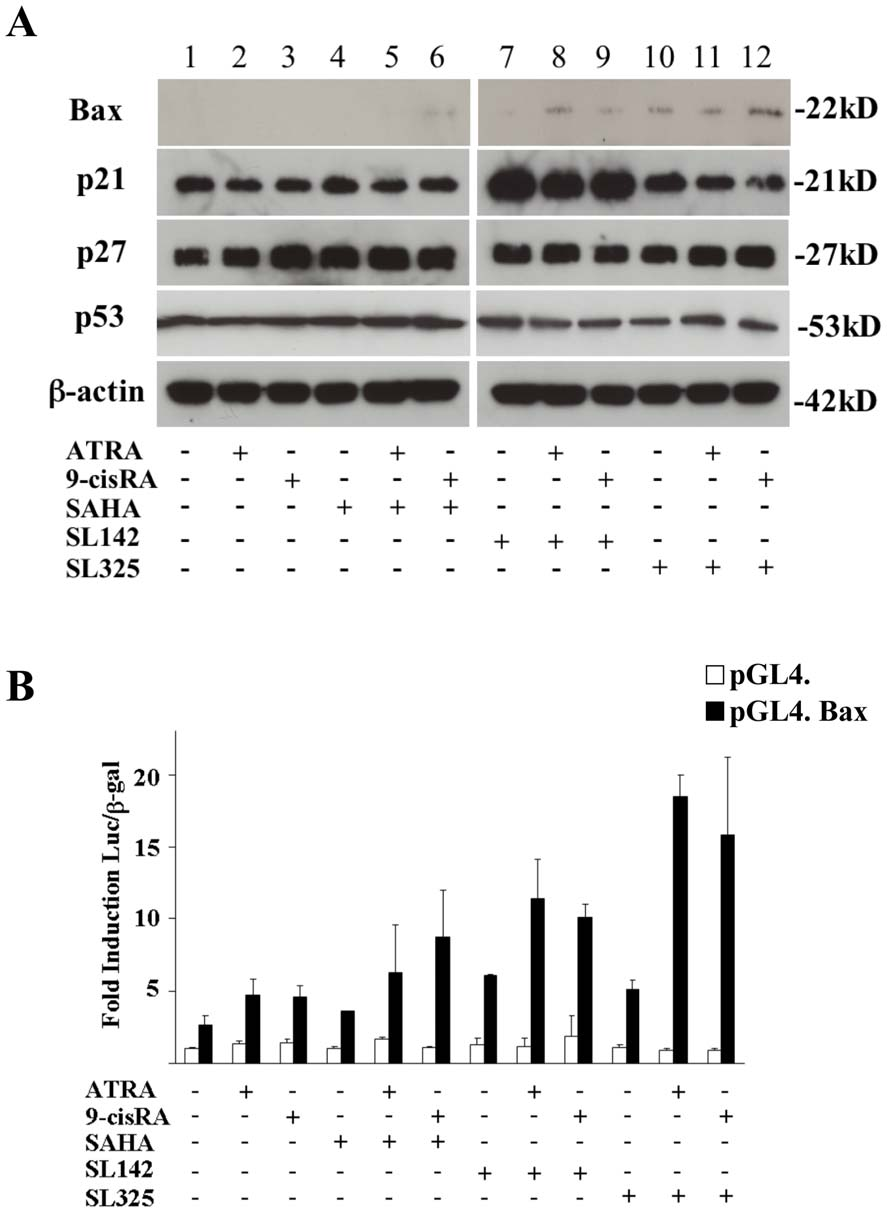
The combination treatment of retinoic acids and/or SL142 or SL325 increased Bax protein expression and the promoter activity in H441 lung cancer cells. **A**. Immunoblot analysis of RARα, RARβ, RXRα and RXRβ expressions after retinoic acids and/or SL142 or SL325 treatment in H441 lung cancer cells. Human lung cancer cells were harvested 24 hours after treatment with ATRA or *9-cis* RA (2.5 µM) or/and SAHA, SL142 or SL325 (0.5 µM). β-actin is shown as control. **B**. Transient transfection reporter assays in H441 cells with pGL4. or pGL4.Bax (2 µg), plus pCMV. β-galactosidase (2 µg) after retinoic acids (2.5 µM) and/or SL142 or SL325 (0.5 µM) retinoic acids treatment. Results are indicated as Fig. 6A. *, significant difference from the promoter activity generated by pGL4.Bax after ATRA or *9-cis* RA (2.5 µM) or SAHA, SL142 or SL325 (0.5 µM) (p<0.05).

In order to investigate the mechanism of the antitumor effect induced by the combination treatment of ATRA or *9-cis* RA and SL142 or SL325, we analyzed the expression of p21, p27 and Bax in H441 lung cancer cells. As shown in Fig. 6A, p21 expression was significantly increased after SL142 treatment (Fig. 6A, lane 7–9). After 9-cisRA, SAHA treatment or combination treatment with SAHA and retinoids increased p27 expression (Fig. 6A, lane 1–6) as reported by others [16], however no significant change was seen in p27 expression after SL142 or SL325 treatment or the combination treatment of SL142 or SL325 and retinoids (Fig. 6A, lane 7–12). Importantly, Bax expression was increased 24 hours after SL142 or SL325 treatment (Fig. 6A, lane 7, 10) and ATRA or *9-cis* RA enhanced the expression (lane 8, 9, 11,12) in H441 cells. The combination treatment of *9-cis* RA and SL325 also increased Bax expression (lane 6). Next, we analyzed Bax promoter activity after the combination treatment using the luciferase construct containing the Bax promoter region: pGL4. Bax. As shown in Fig. 6B, activation of the Bax promoter remained (3.58– to 6.07-fold) after a single treatment of HDAC inhibitors or retinoic acids in H441 lung cancer cells. Importantly, SAHA enhanced Bax promoter activity induced by ATRA or *9-cis* RA (6.27– to 8.74-fold) and SL142 and SL325 enhanced even more (10.14– to 18.44-fold). These results suggest that the combination treatment of SL142 or SL325 and ATRA or *9-cis* RA can enhance the transcriptional activity of the Bax promoter and induce Bax expression in H441 lung cancer cells.

## Discussion

Lung cancer is the most common cause of cancer related deaths worldwide [23]. Treatment of lung cancer with chemotherapy has prolonged survival, however, clinical benefits with these agents is restricted to patients in otherwise good health and those in early stages. But all chemotherapy treatments also have undesirable side effects for the patient [24]. Recently, small molecules have been developed to treat lung cancer in patients carrying such mutated genes as EGFR and EML4-ALK fusion genes. These small molecules are designed to target the kinase domains of the mutated genes and inhibit their activity [25], [26]. However, such small molecule therapy is not suitable for most lung cancer events, including cancer caused by a mutated KRAS gene [27]. Clearly, further investigation of newer, tolerated agents with novel mechanisms of action is urgently needed in order to improve the duration and quality of life for this large population of patients.

Nonselective inhibitors of human histone deacetylases (HDAC) are reported to have antitumor activity *in vivo*, and several of them are under clinical trials [9], [28], [29], [30], [31], [32], [33], [34], [35], [36]. The first of these, SAHA has been approved for treatment of cutaneous T-cell lymphoma and it has also been reported that SAHA has an anti-tumor effect against lung cancer cells [37], [38]. Previously, we developed novel amide-bearing hydroxamic acid derivatives as class-selective HDAC inhibitors termed SL142 and SL325 (Fig. 1A) and reported that these small molecules show more significant HDAC1, HDAC4 and HDAC6 inhibitory activity in human prostate cancer cells LNCaP than SAHA [17]. The half maximal inhibitory concentrations (IC50s) of SL-142 against HDAC1, HDAC4 and HDAC6 are 69±5 nM, 53±2 nM, and 260±70 nM. The IC50s of SL-325 against HDAC1, HDAC4 and HDAC6 are 120±4 nM, 65±4 nM, and 310±50 nM. In contrast the IC50s of the clinically used HDAC inhibitor suberoylanilide hydroxamic acid ZolinzaTM against HDAC1 and HDAC4 are much higher at 290±50 nM, 340±30 nM, and comparable to HDAC6 at 200±10 nM. Therefore, these compounds exhibit more potent inhibitory activity on HDAC1 and HDAC4 and comparable HDAC6 inhibitory activity as compared to the clinically used HDAC inhibitor suberoylanilide hydroxamic acid (ZolinzaTM) [18], [19].

In the present study, SL142 and SL325 showed more significant HADC inhibitory activity (HAT activity) in H441 lung cancer cells (Fig. 1B) and suppressed tumor viability in H441 and A549 lung cancer cells than SAHA (Fig. 1C), indicating that SL142 and SL325 are more effective HDAC inhibitors than SAHA. In our previous report, SL142 and SL325 showed significant p21 promoter activity and cytostatic activity in prostate cancer cells LNCaP [17]. However, in this study, these small molecules also induced significant caspase-3 activity and subG_0_/G_1_ population in H441 and A549 lung cancer cells. These results suggest that SL142 and SL325 could induce apoptosis in lung cancer.

Retinoic acids modulate normal, premalignant, and malignant cell phenotypes by changes in gene expression that are mediated through binding to two classes of nuclear hormone receptors, the RARs and the RXRs. ATRA binds RAR with high affinity but does not bind to RXR, whereas *9-cis* RA is a ligand that binds and transactivates both RARs and RXRs [39], [40]. Retinoic acids regulate differentiation in airway epithelium and suppress carcinogenesis for lung cancer and head and neck cancer [41], [42], [43]. Importantly, it has been reported that the combination treatment of HDAC inhibitors and retinoic acids increased anti-tumor activity in malignant cells [44], [45], [46], [47]. Furthermore, Epping et al. reported that the retinoic acid receptor (RAR) pathway is targeted by HDAC inhibitor and that the antitumor effect of HDAC inhibitor in tumor cells is, in part, through derepression of retinoic acid [48]. In our study, the combination treatment of SL142 or SL325 with these retinoic acids synergically increased apoptosis and suppressed colony formation in the cells (Fig. 3A, 3B, 4A and 4B). Moreover, transcriptional activity generated by RARE after the combination treatment was much higher than that of a single treatment (Fig. 5A). RAR is known to form a heterodimer with RXR and binds to a retinoic acid responsive element (RARE) [49], [50]. However, expression of these receptors was not increased after the treatment in H441 lung cancer cells (Fig. 5B)[45], [51]. Both retinoic acids and HDAC inhibitors have been reported to activate cell cycle or apoptosis related genes and induce cell differentiation or apoptosis in cancer cells [52], [53], [54], [55], [56], [57], [58], [59], [60], [61]. In our report, expression of p21 was clearly increased after SL142 treatment in H441 lung cancer cells and more importantly, Bax expression was increased after the combination treatment of HDAC inhibitors and retinoic acids. Expression of p27 was not changed after SL142 or SL325 and/or retinoid treatment[16]. The promoter of the p21 gene contains a functional RARE [62] and this is consistent with our results (Fig. 5A). However, p21 was originally identified as a molecule that regulates the transition from the G1 phase to the S phase of the cell cycle and overexpression of p21 has been shown to induce differentiation in various cells rather than induce caspase-3 activity and apoptosis [63], [64], [65], [66]. On the other hand over expression of Bax induces caspase-3 activity and apoptosis in many kinds of cells [67], [68], [69]. In this study the combination treatment of SL325 and retinoic acids significantly induced cell death in H441 lung cancer cells. Nevertheless the combination treatment hardly increased p21 expression (Fig. 6A). As shown in Fig. 6A, p53 expression was not altered after the treatment and H441 non-small lung cancer cells carry mutated p53. These results suggest that up regulation of Bax may have a crucial role in the antitumor effect of the combination treatment of SL142 or SL325 and ATRA or *9-cis* RA in H441 lung cancer cells [54] and Bax was up regulated in p53 independent pathway. As shown in Fig. 6A, p53 expression was not altered after the treatment and H441 non-small lung cancer cells carry mutated p53. On the other hand, as shown in Fig. 1C and Fig. 2A, H1299 non-small cell lung cancer cells were less sensitive to SL142 and SL325 compared to H441 and A549 cells and the combination effect was not seen in the cells. For example, H441 and A549 have KRas mutations while H1299 does not. However, we do not know yet which genetic types of NSCLC are responsive to this combination therapy [70], [71]. Further investigation is needed to determine which kinds of lung cancer can be targeted by the combination treatment of SL142 or SL325 and retinoids. We are thus planning to study the applicability of combination therapy using a larger number of cell lines. However, the present study suggests that the combination of retinoic acids and these new kinds of HDAC inhibitors might be a promising approach for treating lung cancer.
